# A glucagon-like peptide-1 analog, liraglutide, ameliorates endothelial dysfunction through miRNAs to inhibit apoptosis in rats

**DOI:** 10.7717/peerj.6567

**Published:** 2019-03-06

**Authors:** Qian Zhang, Xinhua Xiao, Jia Zheng, Ming Li

**Affiliations:** Key Laboratory of Endocrinology, Ministry of Health, Department of Endocrinology, Peking Union Medical College Hospital, Peking Union Medical College, Chinese Academy of Medical Sciences, Beijing, China

**Keywords:** Endothelial function, Diabetes, Apoptosis, Liraglutide, miRNA

## Abstract

**Background and Aims:**

Many studies have revealed that glucagon-like peptide-1 has vasoprotective effects. In this study, we investigated whether liraglutide suppressed endothelial dysfunction and explored the mechanism involved.

**Methods:**

Experimental diabetes was induced through combined high-fat diet administration and intraperitoneal streptozotocin injections. Rats were randomly divided into the following four groups: control, diabetes, diabetes + a low liraglutide dose (0.2 mg/kg/d), and diabetes + a high liraglutide dose (0.4 mg/kg/d). Endothelial function and metabolic parameters were measured after 8 weeks of treatment. miRNA arrays were analyzed to identify the differentially expressed miRNAs.

**Results:**

We found that liraglutide significantly improved aortic endothelial function in diabetic rats. Liraglutide inhibited miR-93-5p, miR-181a-5p and miR-34a-5p expression, and activated miR-26a-5p expression. miRNA mimic transfection experiments indicated negative relationships between miR-93-5p, miR-181a-5p, miR-34a-5p, and miR-26a-5p and Sirt1, Creb, Bcl-2, and Pten expression, respectively. Moreover, liraglutide increased Sirt1, Creb, and Bcl-2 expression levels and reduced Pten expression level.

**Conclusion:**

Our results demonstrate the role of key miRNAs in the liraglutide-mediated regulation of endothelial cell function in diabetic rats.

## Introduction

The worldwide prevalence of diabetes has substantially increased, and diabetes is now recognized as a risk factor for cardiovascular events (Ogurtsova et al., 2017). Chronic hyperglycemia induces a high incidence of cardiovascular diseases through several biochemical and physiological pathways (Capes et al., 2000). Hence, patients with diabetes have a higher risk of atherosclerotic vascular diseases (Capes et al., 2000). Diabetes-induced macrovascular diseases are the leading cause of morbidity and mortality in aging people (Li et al., 2016).

MicroRNAs are a series of small non-coding RNAs that are no more than 30 nucleotides long. In most species, the miRNA sequence is perfectly conserved. However, miRNAs have different functions and target different genes in various tissues, species, and physiological conditions. In general, miRNAs bind to the 3′UTR of target genes, thus affecting the post-transcription level of the protein encoded by the gene (Bartel, 2004). miRNA is also a primary regulator in the cardiovascular system (Latronico et al., 2008; Quintavalle, Condorelli & Elia, 2011). The expression levels of some miRNAs, such as miR-126, miR-92a, and miR-21, are specifically increased in vascular endothelial cells. For example, miR-126 regulates the angiogenic ability of endothelial cells (Fish et al., 2008; Wang et al., 2008), and miR-92a modifies cardiac function in mice with myocardial infarction (Bonauer et al., 2009). In smooth muscle cells, miR-21 accelerates cell proliferation through its targets, including tropomyosin 1, phosphatase and tensin homologue on chromosome 10 (*Pten*), and Bcl-2 (Ji et al., 2007; Wang et al., 2011).

Glucagon-like peptide-1 (GLP-1) is generated from intestinal L-cells that are orally stimulated by glucose loading. GLP-1 participates in glucose-stimulated insulin release and maintains blood glucose homeostasis. However, GLP-1 is rapidly degraded by dipeptidyl peptidase-4 (DPP-4). Since GLP-1 has very short half-life (<2 min after intravenous administration), it is not suitable for a practical option for exogenous therapy (Plamboeck et al., 2005). Therefore, liraglutide, a GLP-1 analog, that is, rarely degraded by DPP-4, was approved for diabetes treatment by the US Food and Drug Administration in 2010. Liraglutide is 97% amino acid homology to native GLP-1. The difference between liraglutide and native GLP-1 is acylation of the lysine reside at position 26 with a hexade-canoyl-glutamyl side chain, and a single lysine-to-arginine amino acid substitution at position 34. Like native GLP-1, liraglutide can activate GLP-1 receptor (Plum, Jensen & Kristensen, 2013). Meanwhile, liraglutide is non-covalently bound to serum albumin, thus it has decreased rate of elimination (Malm-Erjefalt et al., 2010). An increasing amount of evidence shows that GLP-1-based therapies modify cardiovascular function, independent of blood glucose (Bao et al., 2011; Bhashyam et al., 2010). Endothelial dysfunction plays a key role in the early stage of atherosclerosis in diabetes (Chakravarthy et al., 1998). However, little is known regarding liraglutide-induced miRNA actions on endothelial function in diabetic rats.

Thus, in this study, we established a diabetic rat model and applied miRNA microarray and bioinformatics analysis to determine whether liraglutide attenuates vascular dysfunction through miRNA and to explore the underlying mechanism responsible for these effects.

## Materials and Methods

### Animal treatments and diets

Five-week-old male Sprague–Dawley rats (150–180 g) were purchased from the Institute of Laboratory Animal Science, Chinese Academy of Medical Sciences, and Peking Union Medical College (SCXK-2014-0013; Beijing, China). All procedures were performed in accordance with the Guide for the Care and Use of Laboratory Animals published by the US National Institutes of Health (NIH publication No. 85-23, revised 1996) and were approved by the Animal Care Committee of the Peking Union Medical Hospital Animal Ethics Committee (Project XHDW-2015-0051, February 15, 2015). All efforts were made to minimize suffering, and all rats were housed in an environment with controlled temperature (21–23 °C), humidity (50–60%), and light-dark (12:12 h) cycle. After a 1-week acclimatization period, a normal diet (% kcal: 10% fat, 20% protein, and 70% carbohydrate; 3.85 kcal/g) was provided to normal control rats (*n* = 6), and a high-fat diet (% kcal: 45% fat, 20% protein, and 35% carbohydrate; 4.73 kcal/g, Research Diet, New Brunswick, NJ, USA) was provided to the other rats (*n* = 18). After 4 weeks, rats fed the high-fat diet were intraperitoneally injected with streptozotocin (STZ, 30 mg/kg body weight) to develop the induced type 2 diabetes rodent model (Skovso, 2014). The diabetes model was considered successful when rats had fasting blood glucose (FBG) levels exceeding 11.1 mmol/L. Then, diabetic rats were randomly divided into the following three groups (*n* = 6 per group): diabetes + vehicle group (DM, *n* = 6), low liraglutide dose (LL, *n* = 6), and high liraglutide dose (HL, *n* = 6). According previous reports (Hoang et al., 2015; Yang et al., 2018), LL and HL groups were treated with liraglutide at 0.2 and 0.4 mg/kg/d by daily subcutaneous injections for 12 weeks, respectively. Liraglutide doses were based on human dosages adjusted for rodent body weight (Larsen et al., 2008; Sturis et al., 2003). The diabetes + vehicle and control groups were injected with the same volume of normal saline. In addition, the DM, LL, and HL groups were maintained on the high-fat diet through the end of the experiment. At the end of the experiment, the rats were fasted overnight and then anesthetized. Blood samples were obtained from the abdominal aorta. Then, the rats were sacrificed by decapitation. Thoracic aortas were quickly removed, and some of the thoracic aortas were placed in Krebs solution (120 mmol/L NaCl, 4.7 mmol/L KCl, 1.18 mmol/L KH_2_PO_4_, 2.25 mmol/L CaCl_2_, 24.5 mmol/L NaHCO_3_, 1.2 mmol/L MgSO_4_·7H_2_O, 11.1 mmol/L glucose, 0.03 mmol/L EDTA) that was aerated with 95% O_2_ and 5% CO_2_. The aortas were cut into three-mm-long rings for vascular reactivity assays. The remaining aortas were frozen in liquid nitrogen and stored at −80 °C for miRNA arrays and quantitative PCR assays.

### Oral glucose tolerance test

Rats were fasted overnight and were then administered glucose (two g/kg) by gavage (Andrikopoulos et al., 2008; Islam et al., 2009). After glucose loading, blood samples were collected from the tail at 30, 60, and 120 min. Blood glucose levels were measured by a Bayer Contour TS Glucometer (Hamburg, Germany). The blood glucose area under the curve (AUC) was calculated by the linear trapezoid method (Zhang et al., 2016).

### Measurement of fasting serum insulin and homeostasis model assessment of insulin resistance

Serum insulin was determined by ELISA (Millipore, Bellerica, MA, USA). To assess insulin resistance status, homeostasis model assessment of insulin resistance (HOMA-IR) was calculated by FBG (mmol/L) × fasting serum insulin (μIU/mL)/22.5 (Antunes et al., 2016).

### Vascular reactivity assay

Vascular reactivity was measured as described previously (Matsumoto et al., 2014). Thoracic aortas were cut into three-mm rings in Krebs solution aerated with 95% O_2_ and 5% CO_2_ at 37 °C. The rings were pre-contracted with phenylephrine to produce maximal contraction. Then, increasing concentrations of acetylcholine (ACh) were added to the solution to obtain a cumulative concentration-response curve. Isometric tension was recorded using on a BL-410 biological function system (Chengdu Tai Meng Science and Technology Co., Ltd., Chengdu, China).

### RNA extraction and microarray hybridization

To identify key miRNAs modified by liraglutide, we analyzed miRNA microarrays with aorta RNA from the DM and HL groups. RNA from the aorta was extracted using a mirVana^TM^ RNA Isolation Kit (Ambion, Sao Paulo, SP, Brazil). High quality RNA was reverse transcribed into cDNA. cRNA followed by double-stranded cDNA were produced. cDNA was labeled by biotin, and then hybridized to an Affymetrix Multispecies miRNA 4.0 array (Affymetrix Technologies, Santa Clara, CA, USA). Data normalization and analysis were performed with Expression Console software (version 1.4.1, Affymetrix Technologies, Santa Clara, CA, USA). Calculated *P*-values were based on Student’s *t*-tests. miRNAs with fold changes >1.5 and *P*-values < 0.05 were considered differentially expressed miRNAs. A heat map of differentially expressed miRNAs was drawn by using TIGR MeV (MultiExperiment Viewer) software (http://mev.tm4.org/) (Saeed et al., 2006). The microarray raw data were submitted to the Gene Expression Omnibus repository (GSE102198). The validated targets of differentially expressed miRNAs were searched in miRTarBase database version 6.0 (http://mirtarbase.mbc.nctu.edu.tw/, released September 2015) (Chou et al., 2016).

### qRT-PCR for miRNA expression analysis

RNA was isolated as previously described. Reverse transcription was performed by using a miScript^TM^ reverse transcription kit (Qiagen, Hilden, Germany). For miRNA expression, qRT-PCR was performed by using a TaqMan PCR kit (Applied Biosystems, Foster City, CA, USA) and an ABI 7700 system (Applied Biosystems, Foster City, CA, USA). The reaction conditions were as follows: 10 min at 95 °C, followed by 45 cycles of 15 s at 95 °C, and 60 s at 60 °C. miRNA expression levels were normalized to U6 expression.

### qRT-PCR for target gene analysis

qRT-PCR for target genes was performed by using a SYBR Green kit (Applied Biosystems, Foster City, CA, USA). PCR was carried out on an ABI 7700 system (Applied Biosystems, Foster City, CA, USA) using the following reaction conditions: 15 min at 95 °C, followed by 40 cycles of 15 s at 95 °C and 60 s at 64 °C. All gene expression levels were normalized to CypA (cytochrome P450 A) expression. The primers are listed in Table 1.

**Table 1:** Oligonucleotide sequences for qPCR analysis.

Gene symbol	Genebank ID	Forward primer	Reverse primer	Annealing temperature (°C)	Product size
*Bcl2*	NM_016993	GGGATGACTTCTCTCGTCGC	TGACATCTCCCTGTTGACGC	65	200
*Creb1*	NM_031017	GCAGTGACTGAGGAGCTTGT	TGAGCTGCTGGCATGGATAC	65	169
*Pten*	NM_031606	CCAGTCAGAGGCGCTATGTA	TCCGCCACTGAACATTGGAA	64	122
*Sirt1*	XM_017601788	AAGGCAGACAATTTAATGGGGT	ATCGAACATGGCTTGAGGATCT	64	150

**Note:**

*Creb1*, cAMP responsive element binding protein 1; *Pten*, phosphatase and tensin homolog; *Sirt1*, sirtuin.

### Western blotting

Aorta were homogenized in RIPA buffer. Denatured protein (50 μg) were separated on 10% sodium dodecyl sulfate polyacrylamide gel electrophoresis. Polyvinylidene difluoride membranes (Bio-Rad, Hercules, CA, USA) were blocked in Tris-buffered saline buffer with skimmed milk for 30 min, followed by overnight incubation at 4 °C with rabbit anti-PTEN (1:1,000, Cell Signaling Technology, Danvers, MA, USA) or mouse anti-Bcl-2 (1:1,000, Abcam, Cambridge, UK). After washing, membranes were incubated with horseradish peroxidase conjugated secondary antibody for 2 h at room temperature. After incubation, membranes were washed and developed using a chemiluminescence (ECL, Cell Signaling Technology, Danvers, MA, USA) assay. The housekeeping protein β-actin (1:5,000, Abcam, Cambridge, UK) was used for normalization.

### Target gene function and pathway analysis

For miRNA target functional analysis, the database for annotation, visualization, and integrated discovery (DAVID) was used to extract biological features associated with miRNA target gene lists (Dennis et al., 2003). The software performed an enrichment analysis of miRNA target genes with all known Gene Ontology (GO) terms and Kyoto Encyclopedia of Genes and Genomes (KEGG) pathways.

### Cell culture, treatment, and transfection

Human umbilical vein endothelial cells (HUVECs, Life Technologies, Invitrogen^TM^, Carlsbad, CA, USA) were cultured in EGM-2 MV BulletKit medium (Lonza Group Ltd., Basel, Switzerland) in a humidified incubator containing 5% CO_2_ at 37 °C. Based on the miRNA sequence registered in the miRBase database, miRNA mimics, and negative control oligoduplex (Ambion, Thermo Fisher Scientific Inc., Cambridge, MA, USA) were synthesized. HUVECs were transfected with miRNA mimics (50 nM) or negative control using Lipofectamine RNAiMAX transfection reagent (Life Technologies, Carlsbad, CA, USA) for 48 h.

### Data analysis

All data are presented as the mean ± standard deviation (SD). Statistical analysis was determined by Student’s *t*-test and one-way ANOVA, followed by the Tukey–Kramer test for post hoc comparisons using GraphPad Prism software (version 5.0, San Diego, CA, USA).

## Results

### Effect of liraglutide on metabolic parameters in diabetic rats

Metabolic parameters of the experimental rats are summarized in Table 2. Liraglutide significantly decreased body weight after 12 weeks of treatment (*P* < 0.05). Diabetic rats had significant hyperglycemia and higher AUC value in oral glucose tolerance test (OGTT) than that in normal rats (*P* < 0.01). Both low dosage and high dosage of liraglutide treatment could reduce FBG and AUC level in OGTT (*P* < 0.01). Moreover, diabetic rats had higher fasting serum insulin and HOMA-IR than that in normal rats (*P* < 0.01). Liraglutide reduced fasting serum insulin (*P* < 0.05) and HOMA-IR value (*P* < 0.01).

**Table 2:** Metabolic parameters in four groups after treatment.

Parameters	Groups			
	CON	DM	LL	HL
Body weight (g)	512.0 ± 15.5	499.5 ± 9.5	469.8 ± 10.9*^#^	467.8 ± 7.3*^#^
Fasting blood glucose (mmol/L)	5.35 ± 0.41	18.95 ± 2.80**	14.71 ± 2.39**^##^	13.78 ± 2.12**^##^
Fasting serum insulin (ng/mL)	0.89 ± 0.15	1.49 ± 0.33**	0.87 ± 0.21^#^	0.94 ± 0.19^#^
HOMA-IR	4.51 ± 1.07	26.59 ± 6.96**	12.30 ± 4.47**^##^	12.37 ± 4.28**^##^
AUCg (mmol/L/h)	15.61 ± 0.86	46.45 ± 4.94**	38.08 ± 1.83**^##^	32.01 ± 3.29**^##^

**Notes:**

HOMA-IR, homeostasis model assessment of insulin resistance; AUCg, the blood glucose area under the curve; CON, control; DM, diabetes mellitus; LL, low dose of liraglutide; HL, high dose of liraglutide.

Data were presented as mean ± standard deviation (*n* = 6/group). *P < 0.05, **P < 0.01, vs CON; ^#^*P* < 0.05, ^##^*P* < 0.01 vs diabetic.

### Effect of liraglutide on endothelial function in diabetic rats

To evaluate the effect of liraglutide on endothelial function in diabetic rats, endothelium-dependent vasodilation was examined. As shown in Table 3, diabetic rats had significantly impaired endothelium-dependent vasodilation in response to ACh, compared with that in normal rats (*P* < 0.01). Liraglutide treatment ameliorated the impairment of endothelium-dependent vasodilation (*P* < 0.01).

**Table 3:** Vasorelaxation responses (%) to ACh of thoracic aortic rings after treatment.

Groups	ACh (mol/L)						
	1 × 10^−10^	1 × 10^−9^	1 × 10^−8^	1 × 10^−7^	1 × 10^−6^	1 × 10^−5^	1 × 10^−4^
CON	89.8 ± 1.8	83.4 ± 2.4	70.5 ± 3.6	52.5 ± 5.0	33.3 ± 5.5	20.9 ± 5.2	13.5 ± 5.1
DM	91.1 ± 3.6	83.5 ± 1.1	69.1 ± 4.5	57.7 ± 3.4*	40.5 ± 2.5**	28.7 ± 1.3**	21.2 ± 2.6**
LL	91.0 ± 1.5	83.2 ± 3.6	70.9 ± 3.8	48.4 ± 4.3^##^	33.7 ± 3.6^##^	20.4 ± 2.3^##^	13.1 ± 2.3^##^
HL	89.5 ± 2.6	81.0 ± 2.2	71.9 ± 4.0	49.9 ± 2.4^##^	30.7 ± 2.2^##^	17.8 ± 2.2^##^	11.8 ± 2.4^##^

**Notes:**

Data were presented as mean ± standard deviation (*n* = 6). *P < 0.05, **P < 0.01, vs CON; ^##^*P* < 0.01, vs DM.

CON, control; DM, diabetes mellitus; LL, low dose of liraglutide; HL, high dose of liraglutide.

### miRNA array results

**Figure 1:**
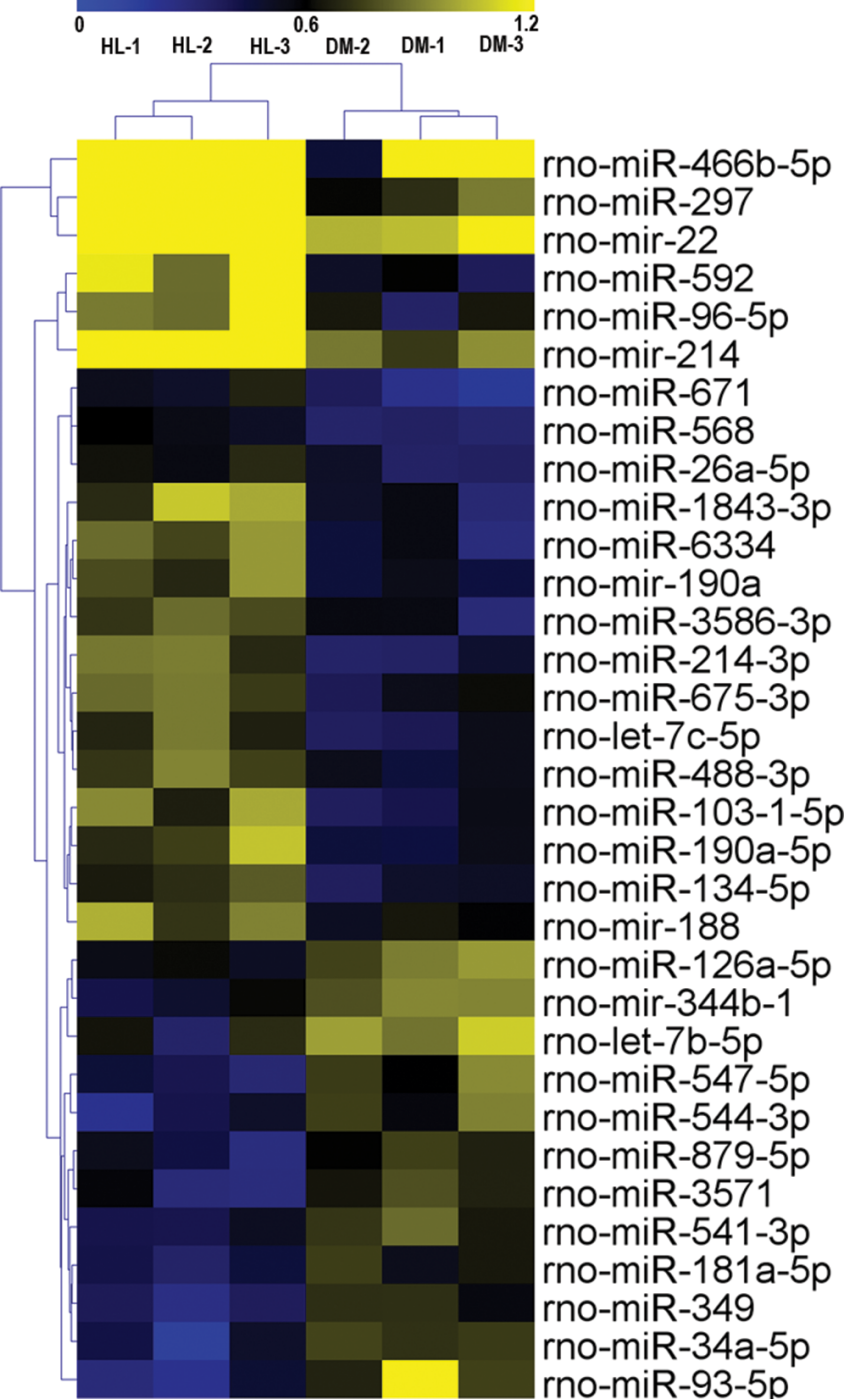
Hierarchical clustering for differentially expressed miRNAs in HL group vs DM group. CON, control; HL, high liraglutide dose.

The miRNA expression profiles of the HL and DM groups were identified by miRNA arrays. The results revealed that 33 miRNAs were significantly changed in the HL group (fold change >1.5, *P* < 0.05, Table 4). Among these 33 miRNAs, 21 miRNAs were upregulated (miR-297, miR-592, miR-671, miR-214-3p, miR-1843-3p, miR-6334, miR-103-1-5p, miR-466b-5p, miR-96-5p, miR-190a-5p, let-7c-5p, mir-22, mir-190a, miR-568, miR-3586-3p, miR-675-3p, mir-214, miR-134-5p, miR-26a-5p, miR-488-3p, mir-188), and 12 miRNAs were downregulated (miR-93-5p, miR-34a-5p, miR-544-3p, miR-349, miR-547-5p, miR-3571, let-7b-5p, mir-344b-1, miR-541-3p, miR-879-5p, miR-181a-5p, miR-126a-5p). Hierarchical clustering of these 33 differentially expressed miRNAs was then performed. Figure 1 shows separate clusters for the diabetes and HL samples.

**Table 4:** Differential expression miRNAs in HL group vs DM group (*P* < 0.05, Fold change >1.5).

miRNA	Fold change	*P*-value	Mature sequence
rno-miR-297	2.817	0.027	AUGUAUGUGUGCAUGUAUGCAUG
rno-miR-592	2.244	0.016	AUUGUGUCAAUAUGCGAUGAUGU
rno-miR-671	2.176	0.020	UCCGGUUCUCAGGGCUCCACC
rno-miR-214-3p	2.129	0.006	ACAGCAGGCACAGACAGGCAG
rno-miR-1843-3p	2.037	0.028	UCUGAUCGUUCACCUCCAUACA
rno-miR-6334	1.991	0.015	CCAGGCUCUCCCAGCUGCCGGC
rno-miR-103-1-5p	1.948	0.023	GGCUUCUUUACAGUGCUGCCUUGU
rno-miR-466b-5p	1.907	0.041	UAUGUGUGUGUGUAUGUCCAUG
rno-miR-96-5p	1.812	0.045	UUUGGCACUAGCACAUUUUUGCU
rno-miR-190a-5p	1.740	0.042	UGAUAUGUUUGAUAUAUUAGGU
rno-let-7c-5p	1.726	0.026	UGAGGUAGUAGGUUGUAUGGUU
rno-mir-22	1.713	0.039	ACCUGGCUGAGCCGCAGUAGUUCUUCAGUGGCAAGCUUUAUGUCCUGACCCAGCUAAAGCUGCCAGUUGAAGAACUGUUGCCCUCUGCCACUGGC
rno-mir-190a	1.687	0.019	UGCAGGCCUCUGUGUGAUAUGUUUGAUAUAUUAGGUUGUUAUUUAAUCCAACUAUAUAUCAAGCAUAUUCCUACAGUGUCUUGCC
rno-miR-568	1.667	0.001	AUGUAUAAAUGUAUACACAC
rno-miR-3586-3p	1.654	0.037	AUACUAGACUGUGAGCUCCUCGA
rno-miR-675-3p	1.602	0.019	UGUAUGCCCUAACCGCUCAGU
rno-mir-214	1.596	0.005	GUCCUGGAUGGACAGAGUUGUCAUGUGUCUGCCUGUCUACACUUGCUGUGCAGAACAUCCGCUCACCUGUACAGCAGGCACAGACAGGCAGUCACAUGACAACCCAGCCU
rno-miR-134-5p	1.592	0.015	UGUGACUGGUUGACCAGAGGGG
rno-miR-26a-5p	1.585	0.021	UUCAAGUAAUCCAGGAUAGGCU
rno-miR-488-3p	1.565	0.012	UUGAAAGGCUGUUUCUUGGUC
rno-mir-188	1.513	0.032	CCCUCUCUCACAUCCCUUGCAUGGUGGAGGGCGAGCUCUCUGAAAACUCCUCCCACAUGCAGGGUUUGCAGGAUGGUGAG
rno-miR-126a-5p	−1.555	0.011	CAUUAUUACUUUUGGUACGCG
rno-miR-181a-5p	−1.597	0.028	AACAUUCAACGCUGUCGGUGAGU
rno-miR-879-5p	−1.639	0.041	AGAGGCUUAUAGCUCUAAGCC
rno-miR-541-3p	−1.668	0.013	AGUGGCGAACACAGAAUCCAUAC
rno-mir-344b-1	−1.729	0.007	AGAGACCUGAUCAGUCAGGCUGCUGGUUAUAUUCCAGGACUUCUCUGGUCCUGGAUAUAACCAAAGCCCGACUGUUAAAUAAAAGUAAGAAUGUGUCU
rno-let-7b-5p	−1.766	0.030	UGAGGUAGUAGGUUGUGUGGUU
rno-miR-3571	−1.837	0.046	UACACACUUCUUUACAUUCCAUA
rno-miR-547-5p	−1.942	0.026	UCACUUCAGGAUGUACCACCCA
rno-miR-349	−1.980	0.007	CAGCCCUGCUGUCUUAACCUCU
rno-miR-544-3p	−1.984	0.044	AUUCUGCAUUUUUAGCAAGCU
rno-miR-34a-5p	−2.079	0.024	UGGCAGUGUCUUAGCUGGUUGU
rno-miR-93-5p	−2.715	0.037	CAAAGUGCUGUUCGUGCAGGUAG

**Note:**

DM, diabetes mellitus; HL, high dose of liraglutide.

### Assay of four differentially expressed miRNAs in the four groups

**Figure 2:**
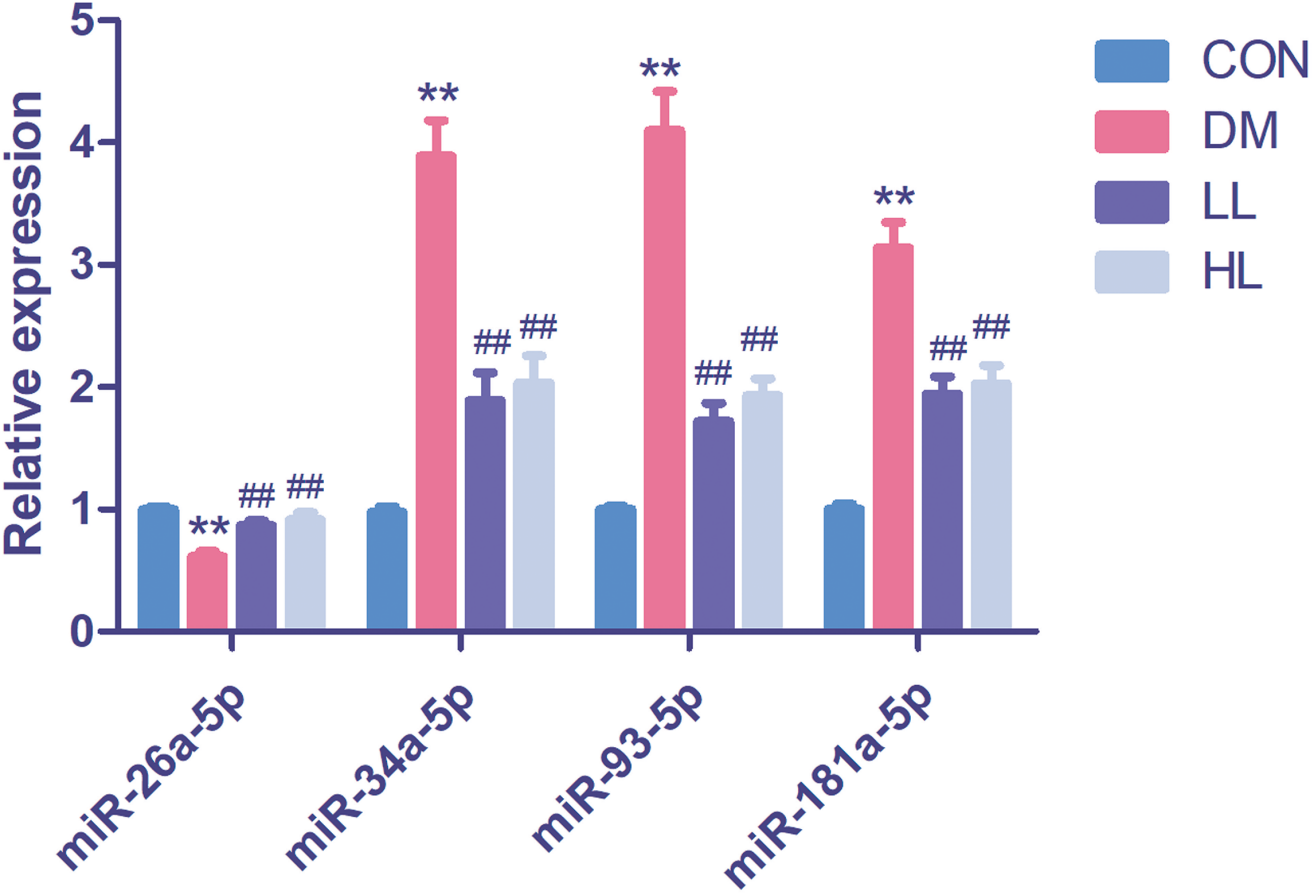
Real time quantification results of the four differentially expressed miRNAs in four groups. Data were presented as mean ± standard deviation (*n* = 6). CON, control; DM, diabetes; LL, low liraglutide dose; HL, high liraglutide dose. **P < 0.01 vs CON group; ^##^*P* < 0.01 vs DM group.

In this experiment, we evaluated four differentially expressed miRNAs in the liraglutide-treated group, as these miRNAs were predicted function to be involved in endothelial function. The four differentially expressed miRNAs were quantified by qPCR to validate the results, as shown in Fig. 2. Among these four miRNAs, all were differentially expressed between the diabetes and HL groups (*P* < 0.01). The results agreed with the miRNA array data.

### Bioinformatic analysis of the targets of 33 differentially expressed miRNAs

**Figure 3:**
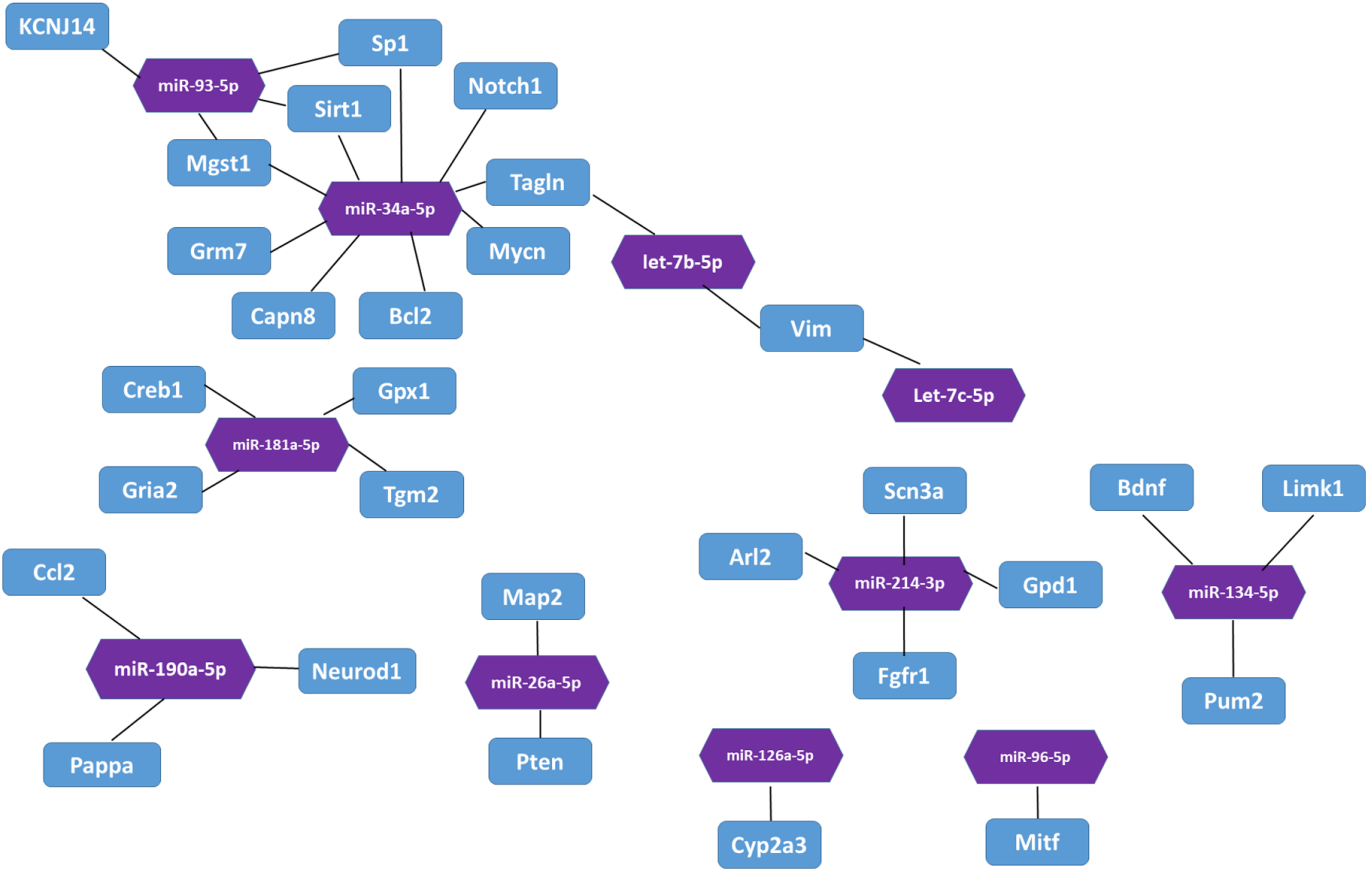
The interaction between miRNA and target genes. Purple hexagon indicates miRNAs and blue rectangles indicates target genes.

In the miRTarBase database, we found 32 experimentally validated target genes from 11 differentially expressed miRNAs (Table 5). Enrichment analysis of the validated target genes was performed to identify the biological processes relevant to the 11 differentially expressed miRNAs in the HL group. GO and KEGG pathway analyses of the targets were performed by using the DAVID database. The results revealed that the 11 miRNAs were involved in eight physiological processes, including the cellular response to tumor necrosis factor, the positive regulation of endothelial cell chemotaxis to fibroblast growth factor, angiogenesis, branching involved in salivary gland morphogenesis, etc. (*P* < 0.05, Table 6). Table 7 lists the top 10 pathways and relevant target genes that were significantly correlated with the 33 miRNAs (*P* < 0.05). The pathways could contribute to the mechanisms of the PI3K-AKT signaling pathway. Figure 3 shows the miRNA-target gene network. Interestingly, several differentially expressed miRNAs regulate genes in the PI3K-AKT signaling pathway.

**Table 5:** Validated target for differentially expressed miRNAs.

miRNA	Regulation	Target genes
rno-miR-214-3p	Up	Fgf16, Gpd1, Arl2, Fgfr1, Scn3a
rno-miR-96-5p	Up	Mitf
rno-miR-190a-5p	Up	Ccl2, Neurod1, Pappa
rno-let-7c-5p	Up	Vim
rno-miR-26a-5p	Up	Map2, Pten
rno-miR-134-5p	Up	Bdnf, Limk1, Pum2
rno-miR-126a-5p	Down	Cyp2a3
rno-miR-181a-5p	Down	Gpx1, Gria2, Tgm2, Creb1
rno-let-7b-5p	Down	Tagln, Vim
rno-miR-34a-5p	Down	Bcl2, Sp1, Tagln, Notch1, Grm7, Capn8, Mgst1, E2f3, Mycn, Sirt1
rno-miR-93-5p	Down	Kcnj14, Sp1, Mgst1, Sirt1

**Table 6:** The genes regulated by the 11 miRNAs related to several biology processes (*P* < 0.05).

Term ID	Term name	Count	*P*-value	Genes	Fold enrichment
GO:0071356	Cellular response to tumor necrosis factor	3	0.002362	GPD1, CCL2, SIRT1	39.2
GO:2000546	Positive regulation of endothelial cell chemotaxis to fibroblast growth factor	2	0.005899	FGFR1, FGF16	323.0
GO:0001525	Angiogenesis	3	0.013624	FGFR1, SIRT1, PTEN	15.9
GO:0060445	Branching involved in salivary gland morphogenesis	2	0.017599	FGFR1, TGM2	107.6
GO:0060045	Positive regulation of cardiac muscle cell proliferation	2	0.027248	FGFR1, NOTCH1	69.2
GO:0002053	Positive regulation of mesenchymal cell proliferation	2	0.031083	FGFR1, MYCN	60.5
GO:0048754	Branching morphogenesis of an epithelial tube	2	0.034903	NOTCH1, MYCN	53.8
GO:0030534	Adult behavior	2	0.040606	GRM7, PTEN	46.1

**Table 7:** The genes regulated by the 11 miRNAs related to several pathways (*P* < 0.05).

Pathway ID	Pathway term	Count	*P*-value	Genes	Fold enrichment
rno05218	Melanoma	5	3.42 × 10^−5^	FGFR1, E2F3, MITF, FGF16, PTEN	24.8
rno05215	Prostate cancer	5	7.62 × 10^−5^	FGFR1, E2F3, CREB1, BCL2, PTEN	20.2
rno05206	MicroRNAs in cancer	6	1.14 × 10^−4^	NOTCH1, E2F3, BCL2, VIM, SIRT1, PTEN	11.3
rno05200	Pathways in cancer	6	0.003477	FGFR1, E2F3, BCL2, MITF, FGF16, PTEN	5.3
rno05161	Hepatitis B	4	0.006636	E2F3, CREB1, BCL2, PTEN	9.7
rno05030	Cocaine addiction	3	0.007296	BDNF, GRIA2, CREB1	22.0
rno05031	Amphetamine addiction	3	0.013088	GRIA2, CREB1, SIRT1	16.2
rno04151	PI3K-Akt signaling pathway	5	0.013482	FGFR1, CREB1, BCL2, FGF16, PTEN	5.0
rno05016	Huntington’s disease	4	0.015083	BDNF, SP1, CREB1, TGM2	7.2
rno05222	Small cell lung cancer	3	0.023206	E2F3, BCL2, PTEN	12.0

### Target gene expression in qPCR

**Figure 4:**
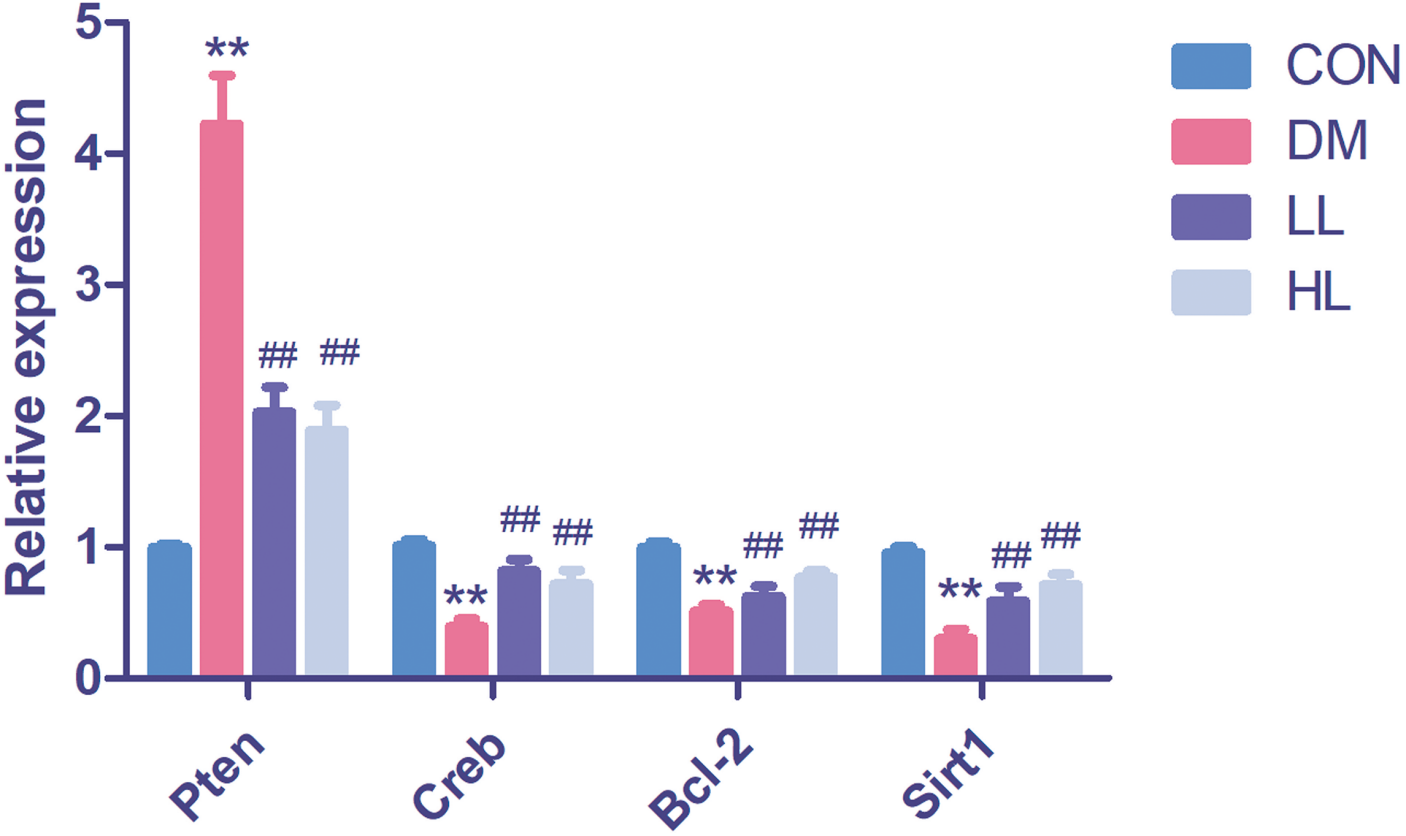
Real time quantification results of the miRNAs target genes in four groups. *Creb1*, cAMP responsive element binding protein 1; *Pten*, phosphatase and tensin homolog; *Sirt1*, sirtuin; CON, control; DM, diabetes; LL, low liraglutide dose; HL, high liraglutide dose. Data were presented as mean ± standard deviation (*n* = 6). **P < 0.01 vs CON group; ^##^*P* < 0.01 vs DM group.

We found that *Pten* expression was significantly upregulated (*P* < 0.01, Fig. 4); however, *Creb1*, *Bcl2*, and *Sirt1* levels were significantly downregulated in the DM group (*P* < 0.01, Fig. 4). Liraglutide reduced *Pten* expression and increased *Creb1*, *Bcl2*, and *Sirt1* expression (*P* < 0.01, Fig. 4).

### Protein expression in western blot

**Figure 5:**
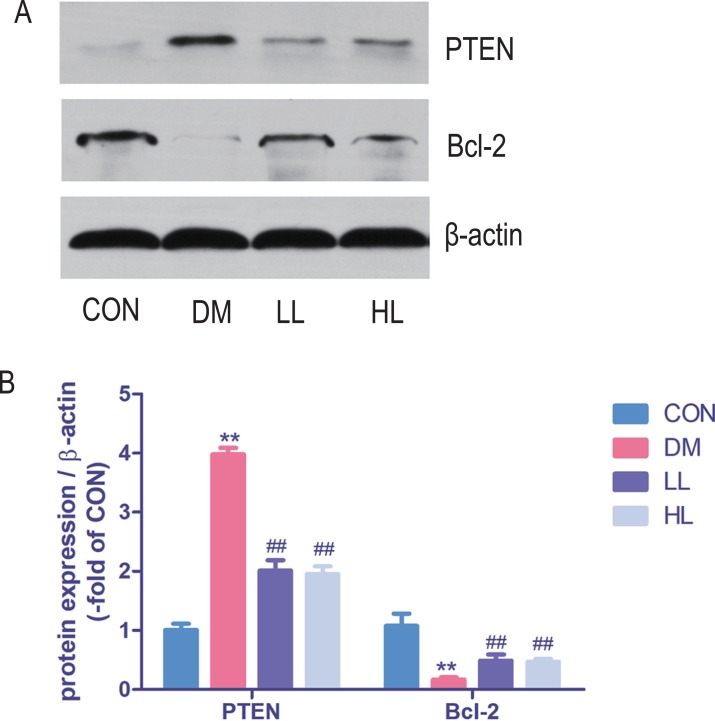
Protein expression levels were detected by Western blot. CON, control; DM, diabetes; LL, low liraglutide dose; HL, high liraglutide dose; PTEN, phosphatase and tensin homolog. Data were presented as mean ± standard deviation (*n* = 6). **P < 0.01 vs CON group; ^##^*P* < 0.01 vs DM group.

Total protein expression of PTEN was significantly higher, while Bcl-2 was lower in DM group than that in CON group (*P* < 0.01, Fig. 5). Liraglutide reduced PTEN expression and increased Bcl-2 expression in aorta (*P* < 0.01, Fig. 5).

### Target gene expression in miRNA mimic-transfected HUVECs

**Figure 6:**
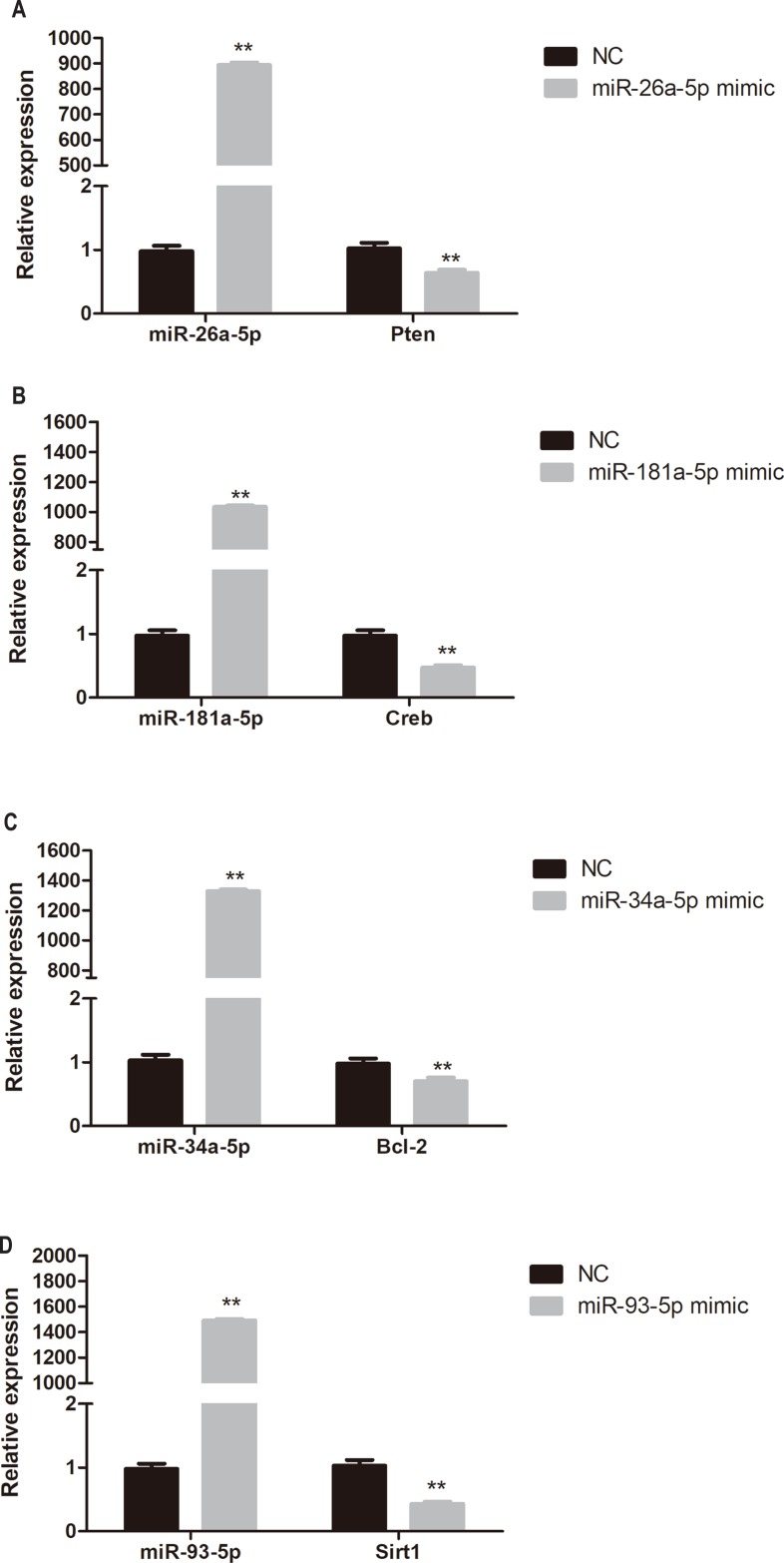
Quantitative PCR analysis of miR-26a-5p, miR-181a-5p, miR-34a-5p, miR-93-5p, *Pten*, *Creb*, *Bcl-2*, and *Sirt1* expression in miR-26a-5p (A), miR-181a-5p (B), miR-34a-5p (C), miR-93-5p (D) mimic transfected HUVECs. Data were presented as mean ± standard deviation (*n* = 6). **P < 0.01 vs the NC group. *Creb*, cAMP responsive element binding protein; NC, negative control; *Pten*, phosphatase and tensin homologue on chromosome 10; Sirt1, Sirtuin 1.

To determine whether miR-26a-5p, miR-181a-5p, miR-34a-5p, and miR-93-5p negatively regulate their target genes in vitro, HUVECs were transfected with respective miRNA mimics. *Pten*, *Creb*, *Bcl-2*, and *Sirt1* expression was significantly reduced in miR-26a-5p, miR-181a-5p, miR-34a-5p, and miR-93-5p mimic transfected HUVECs, respectively (*P* < 0.01, Fig. 6).

## Discussion

In our study, we showed that liraglutide treatment significantly ameliorated blood glucose and insulin resistance status in diabetic rats. Then, we used aortic rings to evaluate ACh-induced endothelium-dependent vasodilation. ACh-induced endothelium-dependent vasodilation was impaired in diabetic rats, while liraglutide augmented endothelial function. Clinical trials prove liraglutide as a potent drug for diabetic patients with cardiovascular disease. The Liraglutide Effect and Action in Diabetes: Evaluation of Cardiovascular Outcome Results trial showed that patients treated with liraglutide had fewer primary outcomes pertaining to non-inferiority, cardiovascular-related death, or all-cause death than patients treated with a placebo (Marso et al., 2016). Many in vitro and in vivo animal research proves the benefit of liraglutide on vascular. In rat branched mesenteric arteries, liraglutide induce significant relaxation compared with vehicle (Bangshaab et al., 2018). In rodent research, liraglutide also inhibits endothelial cell dysfunction in ApoE^−/−^ mouse model (Gaspari et al., 2011).

A previous study indicated the synergistic effect of liraglutide with metformin on endothelial dysfunction through the GLP-1 receptor and the protein kinase A signaling pathway (Ke et al., 2017). In our study, pathway analysis revealed many miRNA targets in the PI3K-AKT pathway. The PI3K-AKT signaling pathway plays various roles in cell biology function, including endothelial cell proliferation. A previous study revealed that PI3K-AKT signaling inhibited human cardiac microvascular endothelial cells (HCMEC) apoptosis and hypoxia/reoxygenation (HR)-induced endothelial dysfunction. Moreover, the PI3K-AKT pathway prevented HR-induced myocardial microvascular endothelial cell apoptosis (Su et al., 2014). Increased levels of Bcl-2, PI3K, and phosphorylated AKT can protect HCMECs from HR injury (Xie et al., 2016). The liraglutide-treated group presented with increased miR-26a-5p expression. In addition, an in vitro experiment also showed that liraglutide increased miR-26a-5p expression in high glucose-exposed HUVECs. PTEN is a miR-26a-5p target genes that can dephosphorylate phosphatidylinositol-3,4,5-triphosphate produced by PI3K, and interrupt AKT production to inhibit the PI3K-AKT pathway (Kang, Lemke & Kim, 2009). Thus, Pten is a primary regulator of the balance between survival and death in many cell lines, including vascular endothelial cells. Pten loss-of-function mutant mice exhibited enhanced activity of 3-phosphoinositide-dependent protein kinase and AKT (Kurose et al., 2001; Stambolic et al., 1998). In addition, Pten expression level was significantly increased in diabetic mouse aortas (Takenouchi et al., 2008). Our results proved that liraglutide reduced high-glucose-induced high expression of Pten. In addition to phosphorylating AKT, PI3K also regulates several other pathways, including the Bcl-2 pathway. Bcl-2 is a major anti-apoptotic protein that prevents cytochrome C release into the cytoplasm (Do et al., 2015; Li et al., 1998; Luo et al., 1998). miRNA mimic and inhibitor transfection demonstrated a negative correlation between miRNA-26a-5p and *Pten* expression. Moreover, we found that miR-181a-5p expression was decreased in the liraglutide-treated group. Creb is a miR-181a-5p target, and we also found that liraglutide treatment enhanced *Creb* expression in the aorta. In addition, miR-181a-5p expression is negatively correlated with *Creb* in vitro. Bcl-2 phosphorylation is followed by Creb serine-133 phosphorylation (Das et al., 2005). We found that liraglutide can reduce miR-34a-5p expression in vivo and in vitro. Inhibition of the miR-34 family by locked nucleic acid-modified antimiR improved systolic function in mice with impaired pathology and function (Bernardo et al., 2012). Bcl-2 is a miR-34a-5p target gene. We further found that *Bcl-2* expression was higher in the liraglutide-treated group than in the diabetes group and that miR-34a-5p and Bcl-2 expression levels were negatively correlated. High glucose levels reduced anti-apoptotic Bcl-2 expression in endothelial cells (Qin et al., 2016). Our result suggests that liraglutide increased Bcl-2 expression in high-glucose exposed HUVECs. Previous studies have shown that antioxidants can activate the Bcl-2 protein to inhibit INS-1 cell apoptosis (Zheng et al., 2015). A previous study found that exendin-4 increases Bcl-2 protein levels in tert-butyl hydroperoxide-treated HUVECs (Wang et al., 2016). Thus, our study showed that the PI3K-AKT signaling pathway was involved in the anti-apoptotic effects of liraglutide in endothelial cells.

Moreover, we found that liraglutide inhibited miR-93-5p expression in diabetic rat aortas. Sirtuin 1 (Sirt1) is target of miR-93-5p (Li et al., 2011) and we found that the expression of Sirt1 increased in liraglutide-treated rat aorta. miRNA mimic transfection revealed that miR-93-5p expression is negatively related to Sirt1 expression in HUVECs. SIRT1 is a type of NAD^+^-dependent histone deacetylase and has various functions in inflammation, aging, and apoptosis (Caito et al., 2010). Sirt1 contributes to vascular protection through the deacetylation of several targets, including FOXO3 (Hwang et al., 2013) and p53 (Nadtochiy et al., 2011). Sirt1 has been previously recognized to protect endothelial cells against hyperglycemia-induced oxidative stress (Kumar et al., 2017) and apoptosis (Wang et al., 2017). Our study demonstrated that liraglutide inhibits miR-93-5p, activating Sirt1 expression to protect endothelial cell function.

## Conclusion

**Figure 7:**
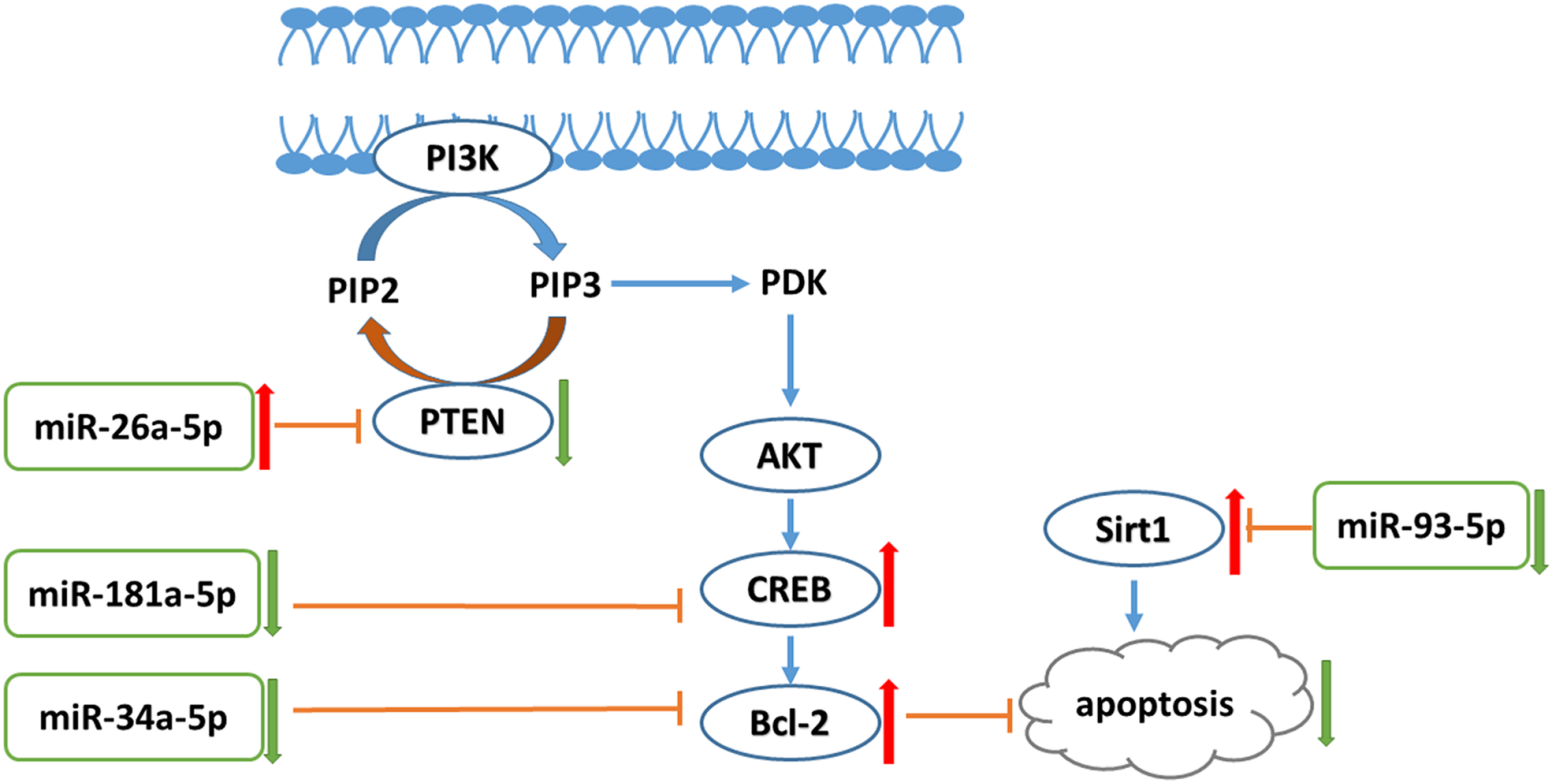
PI3K/AKT-Bcl-2 pathway was involved in the liraglutide treated aorta. Liraglutide increases miR-26a-5p to reduce PTEN thus increasing the levels of PIP3 to stimulate the PI3K-activated signaling cascades. In addition, liraglutide reduces miR-181a-5p and miR-34a-5p to increase the expression of Creb and Bcl-2, thus inhibiting the cell apoptosis. PTEN, phosphatase and tensin homologue on chromosome 10; PI3K, phosphoinositide 3-kinase; PIP2, phosphatidylinositol 4,5-bisphophate; PIP3, phosphatidylinositol (3,4,5)-trisphosphate; PDK, phosphoinositide-dependent kinase-1; Akt, serine/threonine kinase 1; Creb, cAMP responsive element binding protein; Sirt1, Sirtuin 1.

In conclusion, liraglutide can attenuate endothelial dysfunction. Liraglutide activates miR-26a-5p expression and inhibits miR-181a-5p, miR-34a-5p, and miR-93-5p expression in the aorta to inhibit endothelial cell apoptosis through the PI3K-AKT-Bcl-2 activation pathway (Fig. 7). In addition to the transcriptome, these results may provide attractive therapeutic options for targeting the epigenetic mechanisms of endothelial dysfunction via miRNAs.

## Supplemental Information

10.7717/peerj.6567/supp-1 Supplemental Information 1 Raw data of metabolic indexes, miRNA qPCR, and western blot.

## Additional Information and Declarations

### Microarray Data Deposition

The following information was supplied regarding the deposition of microarray data: The microarray raw data were submitted to the Gene Expression Omnibus (GEO) repository (GSE102198).

### Data Availability

The following information was supplied regarding data availability: The raw measurements are available in File S1.
